# Advanced Unilateral Retinoblastoma: The Impact of Ophthalmic Artery Chemosurgery on Enucleation Rate and Patient Survival at MSKCC

**DOI:** 10.1371/journal.pone.0145436

**Published:** 2015-12-28

**Authors:** David H. Abramson, Armida W. M. Fabius, Reda Issa, Jasmine H. Francis, Brian P. Marr, Ira J. Dunkel, Y. Pierre Gobin

**Affiliations:** 1 Department of Surgery, Memorial Sloan Kettering Cancer Center, NewYork, New York, United States of America; 2 Department of Ophthalmology, Weill Cornell Medical College, New York Presbyterian Hospital, New York, New York, United States of America; 3 Department of Pediatrics, Memorial Sloan Kettering Cancer Center, New York, New York, United States of America; 4 Department of Pediatrics, Weill Cornell Medical College, New York Presbyterian Hospital, New York, New York, United States of America; 5 Interventional Neuroradiology, Departments of Radiology, Neurosurgery and Neurology, Weill Cornell Medical College, New York Presbyterian Hospital, New York, New York, United States of America; Massachusetts Eye & Ear Infirmary, Harvard Medical School, UNITED STATES

## Abstract

**Purpose:**

To report on the influence of ophthalmic artery chemosurgery (OAC) on enucleation rates, ocular and patient survival from metastasis and impact on practice patterns at Memorial Sloan Kettering for children with advanced intraocular unilateral retinoblastoma.

**Patients and Methods:**

Single-center retrospective review of all unilateral retinoblastoma patients with advanced intraocular retinoblastoma treated at MSKCC between our introduction of OAC (May 2006) and December 2014. End points were ocular survival, patient survival from metastases and enucleation rates.

**Results:**

156 eyes of 156 retinoblastoma patients were included. Primary enucleation rates have progressively decreased from a rate of >95% before OAC to 66.7% in the first year of OAC use to the present rate of 7.4%. The percent of patients receiving OAC has progressively increased from 33.3% in 2006 to 92.6% in 2014. Overall, ocular survival was significantly better in eyes treated with OAC in the years 2010–2014 compared to 2006–2009 (p = 0.023, 92.7% vs 68.0% ocular survival at 48 months). There have been no metastatic deaths in the OAC group but two patients treated with primary enucleation have died of metastatic disease.

**Conclusion:**

OAC was introduced in 2006 and its impact on patient management is profound. Enucleation rates have decreased from over 95% to less than 10%. Our ocular survival rate has also significantly and progressively improved since May 2006. Despite treating more advanced eyes rather then enucleating them patient survival has not been compromised (there have been no metastatic deaths in the OAC group). In our institution, enucleation is no longer the most common treatment for advanced unilateral retinoblastoma.

## Introduction

Enucleation has always been the most common treatment for both unilateral and bilateral retinoblastoma[1]. Although it has resulted in very good patient outcomes, it is a surgical procedure that leaves a permanent cosmetic reminder and deprives the patient of any possible sight in the eye(s). External beam irradiation was the first treatment technique that allowed clinicians to save an eye–often with useful vision. Introduced more than 100 years ago it was the only treatment that allowed salvaging of eyes with advanced disease. For patients with lower staged disease (Reese-Ellsworth I-III) success rates were high[2]. For advanced eyes (Reese-Ellsworth IV-V), which represented more than 75% of all eyes at presentation, success rates were 20%[3]. Unfortunately external beam irradiation significantly altered the timing and pattern of second cancers resulting in shortened life span. Second cancers in radiated children followed a dose response curve[4], were associated with more cancers “in the field” at a younger age[5] and were especially notable in children who received radiation in the first year of life[6] or in combination with systemic chemotherapy[7]. In an attempt to minimize the use of radiation clinicians worldwide replaced external beam radiation with systemic chemotherapy in the mid 1990s[8]. Although chemotherapy alone was rarely curative, when combined with focal techniques such as laser, cryotherapy or brachytherapy (and occasionally external beam irradiation) many eyes could be salvaged[9]. Eyes with advanced retinoblastoma however were salvaged in fewer than 50% of cases[8] and side effects, including secondary Acute Myeloid Leukemia were worrisome[10].

Reese first did intrarterial chemotherapy via the carotid artery 70 years ago and called it “arterial chemotherapy”[11]. Subsequently the Japanese introduced a technique with a micro balloon; they temporarily occluded the internal carotid artery and injected drug below the balloon near the exit for the ophthalmic artery. This was called “selective ophthalmic artery infusion of chemotherapy”[12].

Ophthalmic artery chemosurgery was introduced by us in 2006 as an alternative to enucleation, external beam irradiation and systemic chemotherapy for advanced retinoblastoma[13]. With this technique (after heparanization) a microcatheter is placed at the ostium of the ophthalmic artery under anesthesia and drug delivered selectively into the ophthalmic artery. This technique was originally called “super selective ophthalmic artery infusion of chemotherapy” but the preferred term is now “ophthalmic artery chemosurgery” (OAC). In both the Japanese and our technique access is via the femoral artery (Reese used the internal carotid artery).

Since the introduction of OAC it has been performed successfully in more than 45 countries and has more than 200 publications in the peer-reviewed literature. Moreover, in a recent survey OAC was the first choice for advanced eyes in the majority or centers worldwide[14]. The purpose of this report is to explain how OAC has changed our practice patterns and report on the ocular survival, enucleation rate and metastatic deaths in our retinoblastoma population.

## Materials and Methods

### Subjects

This is a retrospective chart review of patients treated at Memorial Sloan Kettering Cancer Center (MSKCC) between May 2006 and December 2014 with advanced intraocular unilateral retinoblastoma. Memorial Sloan Kettering Cancer Center’s Institutional Review Board (IRB) has approved this retrospective study; the IRB waiver number is WA0634-14. Written consent has been obtained from next of kin/caregivers for all patients in order to perform enucleations, and OAC treatments and patient records/information was anonymized and de-identified prior to analysis.

Advanced retinoblastoma was defined as either Reese-Ellsworth group “Va” or “Vb” *and* International Classification of Retinoblastoma (ICRb) “D” or “E” (Children’s Oncology Group-COG groups). Patients were treated with either primary enucleation within 30 days of diagnosis at MSKCC (that is, no prior treatment) or OAC. The start date of this series represents the advent of OAC at our institutions. Clinical characteristics including age at presentation, follow-up time, ocular and patient survival and treatment history and metastatic information were collected via the electronic medical record.

Both naïve eyes and eyes that had received prior non-OAC therapy (systemic or intravitreal chemotherapy, external beam or plaque radiotherapy) were included in the analysis, the latter were referred to as “prior treated” eyes.

The trend of enucleations vs OAC was calculated yearly as the total number of eyes undergoing enucleations or OAC as primary treatment once at our institution, divided by the total number of eyes (sum equals 100%). The total number of patients was used to calculate the ratio of patients who underwent OAC vs enucleations. Prism (GraphPad Software, Inc, La Jolla, CA) was used to calculate the significance of the D versus E eyes OAC treated and enucleated in the period between 2006–2009 and 2010–2014 (Fisher’s exact test, two tailed).

### Ocular survival

Statistical analysis was performed with Prism. Kaplan-Meier survival data with the log-rank test were used to evaluate ocular survival, and the Mantel-Cox test was used to compare survival curves. The 95% confidence intervals were used and 48-month ocular survival was reported.

## Results

### Baseline Characteristics

Clinical characteristics of each group are depicted in Table 1. One hundred fifty six patients with advanced unilateral retinoblastoma were identified. Forty nine percent of eyes we treated with OAC had prior therapy elsewhere (mostly intravenous chemotherapy). None of the patients whom we primarily enucleated had prior attempts at therapy.

**Table 1:** Clinical Characteristics of Eyes with Advanced Stage Unilateral Retinoblastoma. Eyes are either treated with OAC or primary enucleation.

Features		OAC	Primary enucleation
Number of patients		96	60
Number of eyes		96	60
Mean Age (Mos.)		26	27
	Median (range)	21 (2–122)	24 (6–94)
Mean follow-up (Mos.)		29	30
	Median (range)	20 (2–98)	26 (1–76)
Family History			
Negative		95 (99%)	58 (97%)
Positive		1 (1%)	2 (3%)

### Enucleation and OAC Trend

**Fig 1:**
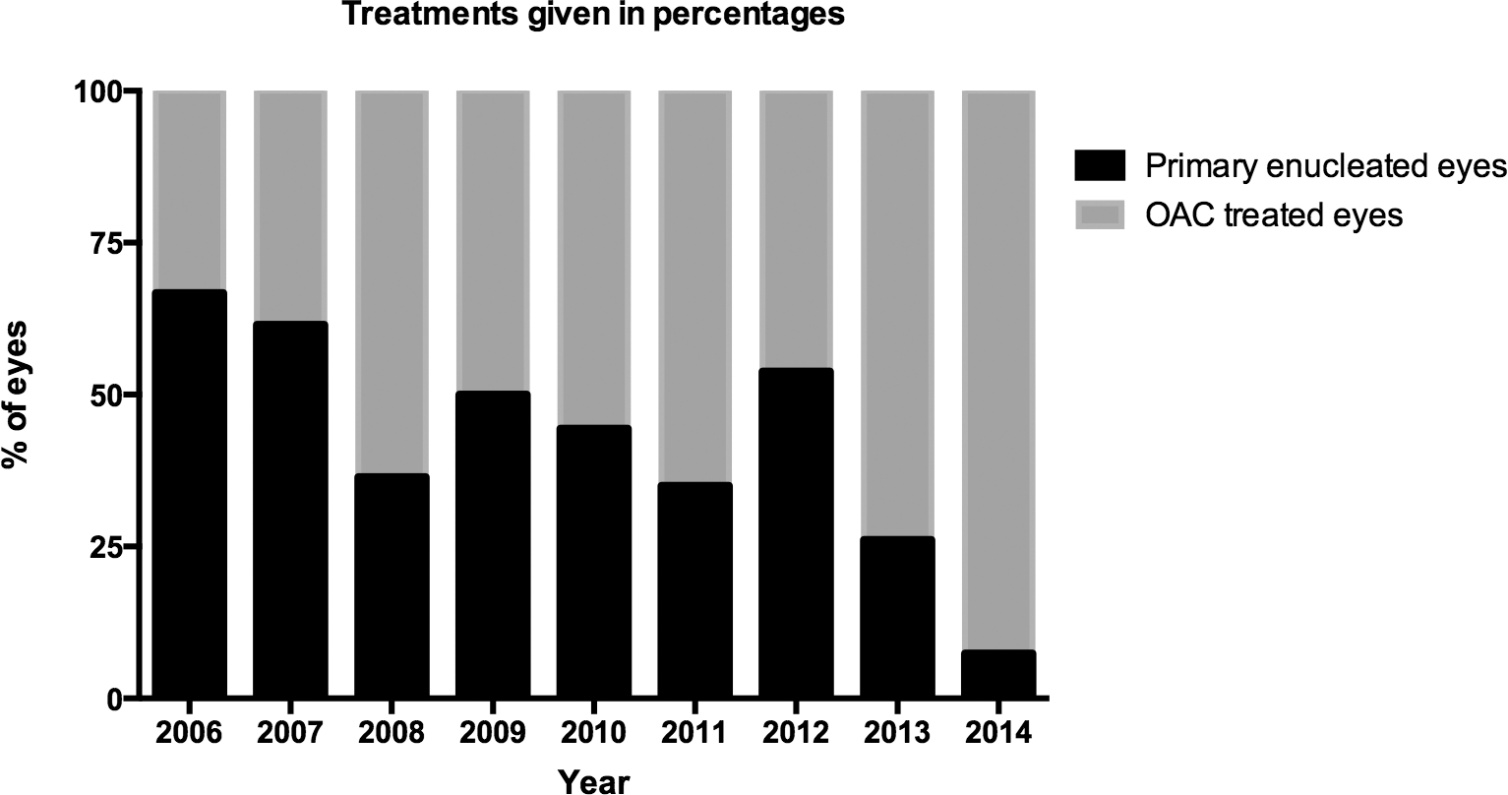
Treatments in unilateral advanced eyes. Stacked bar of graph of the percentage of eyes that were primarily enucleated (black) or treated with OAC (gray) per year.

OAC began in May 2006; in that year 66.7% of eyes with advanced disease were primarily enucleated versus only 7.4% in 2014 (Fig 1). The shift from enucleation towards treatment with OAC between 2006 and 2014 is significant (Fisher’s exact, p<0.0001).

### Total number of advanced eyes seen

**Fig 2:**
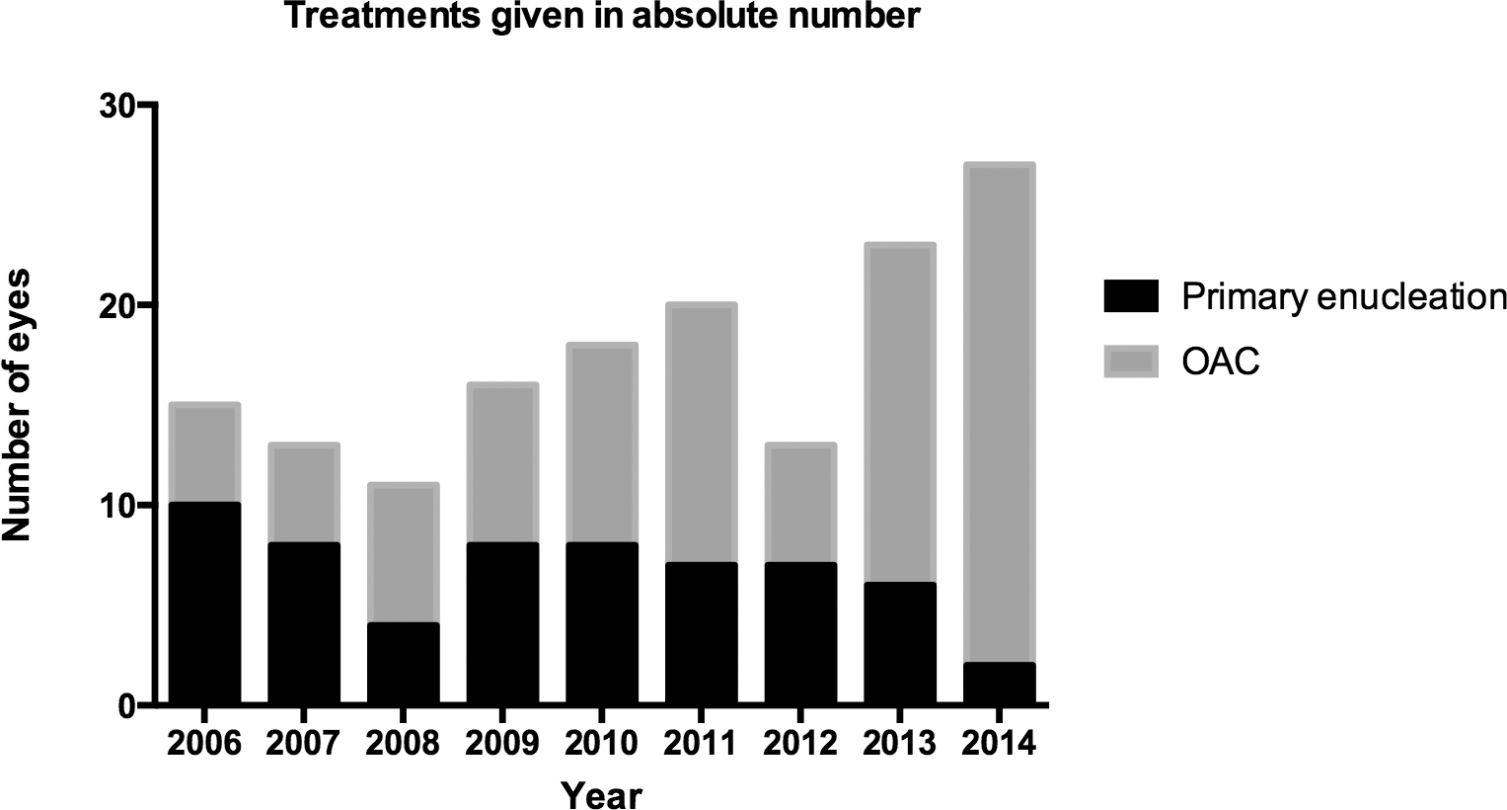
Primary enucleation and OAC treatment in unilateral advanced eyes. The number of eyes that were primarily enucleated (black) or treated with OAC (gray) per year.

Our clinical volume of advanced intraocular disease has grown since 2006. In the years 2006–2009 the total number of unilateral eyes primarily enucleated or OAC treated was only 55 while this volume almost doubled in the years 2010–2014 to 101 eyes (Fig 2).

ICRb groups were compared within these two periods and between OAC and primarily enucleated eyes. The distribution of D versus E eyes changed in time for OAC treated eyes; significantly more E eyes were OAC treated the period 2010–2014 compared to 2006–2009 (Fisher’s exact; p = 0.0016).

For both periods the distribution of the number of treated D versus E eyes was different in OAC treated versus primarily enucleated eyes, relatively more E eyes got primarily enucleated (Fisher’s exact; p<0.0001) (S1 Fig). The treatment choice for D eyes did not change in time (S2A Fig). However, there is a clear shift in treatment choice for E eyes. In the period 2006–2009 only 3% of E eyes were OAC treated whereas in the period 2010–2014 this increased to 50% (Fisher’s exact; p<0.0001) (S2B Fig).

### Ocular Survival

**Fig 3:**
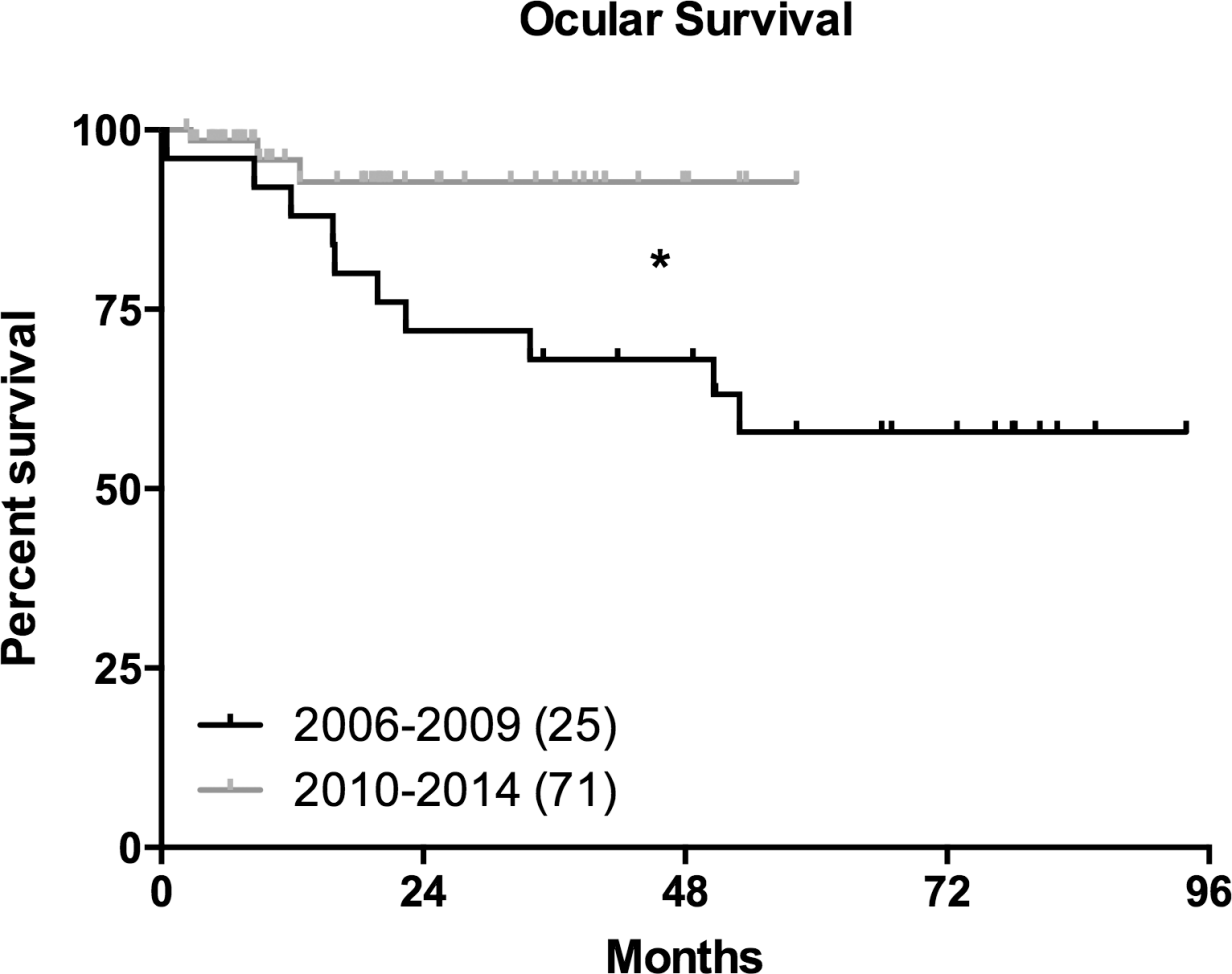
Ocular survival in unilateral advanced eyes treated with OAC. Ocular survival Kaplan-Meier curves for 96 patients, who had been treated with OAC. Patients were divided according to treatment period, 2006–2009 (black) and 2010–2014 (grey). The number of eyes in each group are depicted in the legend, * depicts a significant change; Mantel Cox test p value = 0.023.

Ocular survival of OAC treated eyes was different when the early period of 2006–2009 (n = 25) was compared to the later period of 2010–2014 (n = 71). The 48 month ocular survival was significantly better in the later period (p = 0.0234), respectively 68.0% (95% CI, 46.1%-82.5%) for the 2006–2009 period vs 92.7% (95% CI, 78.1%-97.7%) for the 2010–2014 period (Fig 3). It is difficult to pinpoint confounding factors for this better ocular survival in the more recent period. Because improvement has been steady since 2006 responsible factors may include a learning curve, greater use of more than one chemotherapeutic agent and the use of intravitreal injections of chemotherapy. We began intravitreal injections in September 2012 so improvement in ocular salvage rates between 2006 and 2012 were not attributable to the injections.

### Development of metastasis

In the OAC cohort, three patients (3.1%) developed metastatic retinoblastoma. Each of these patients is disease-free with follow-up of 3, 4 and 5 years after metastasis diagnosis, (total follow up for these patients is respectively 4,7,8 years). Six patients who were primarily enucleated developed metastatic disease (10%) Four of these patients are disease-free with follow-up of 1, 3, 5 and 8 years after metastasis diagnosis, (total follow up for these patients is respectively 1, 3, 5, 9 years). Three of the 6 patients who have developed metastasis after primary *enucleation* had higher risk factors (retrolaminar optic nerve invasion or extensive choroidal invasion) at the time of enucleation. None received adjuvant chemotherapy. When metastasis was diagnosed 5/6 patients received chemotherapy and 1/6 has received irradiation therapy.

Two of these patients died respectively 1 year and 2 years after metastasis diagnosis (total follow up for these patients is respectively 2 and 3 years).

## Discussion

Since our introduction of ophthalmic artery chemosurgery in 2006 it has been replicated in more than 40 countries worldwide, and its advantage over conventional treatments have been emphasized by many authors. It has been successful in avoiding enucleation for eyes with advanced unilateral disease[15], bilateral disease (called “tandem therapy”[16]), eyes with extensive retinal detachment[17,18], eyes with vitreous and subretinal seeding[19,20] for both naive and eyes that progressed with conventional therapy[21,22]. Both single agent and multiple drug regimens are used; the drugs used are Melphalan (most commonly), Carboplatin and Topotecan. This is an outpatient procedure and because of the low doses of drug used has little hematologic consequences (fewer than 2% of patients develop febrile neutropenia or require transfusion of any blood products)[23]. This paper details the impact of this treatment on the management of retinoblastoma at MSKCC.

Virtually every paper, book and teaching guide written in the 20^th^ century about the management of retinoblastoma emphasized that enucleation was the most common treatment performed for unilateral and bilateral retinoblastoma worldwide. For example, Reese and Duke-Elder stated that enucleation was the only option for patients with unilateral disease[1]. Not until the last part of the 20^th^ century were unilateral eyes treated with anything but enucleation[24] and even then *advanced* eyes were rarely treated with anything but enucleation. For bilateral cases the standard management for most of the 20^th^ century was enucleation of the most advanced eye and radiation of the fellow eye[25]. Overall the majority of patients had at least one enucleation, and more than 25% had both eyes removed[26].

The introduction of OAC has reversed this 100-year-old management scheme. In just 9 years we have decreased our enucleation rate for advanced eyes from over 95% to 7.4%. In addition, the ocular survival increased significantly in the period 2010–2014 compared to the first 4 years that OAC was used in our institution. We think that this increased ocular survival might be caused by a number of factors. In May 2006 this was a new procedure, and there has been a learning curve for both the ophthalmologist in managing the disease and the interventional neuro radiologist in doing the procedure. In addition the use of intravitreous injections for eyes with vitreous seeding began during the second time period. Furthermore, in time there has been a shift towards triple therapy and treatment recurrences are now very successfully treated with three cycles of OAC [27]

The decision about enucleation vs. OAC varies from patient to patient because it is a clinical process influenced by family preference and in some cases cost. In general eyes with rubeotic glaucoma, buphthalmos and anterior chamber involvement are enucleated but some families will not accept that so some of these eyes are therefore treated with OAC. The improvement is not due to selection bias, however, as there are more advanced eyes (“E”) in the most recent group (with the highest success rate).

Aziz and colleagues compared the costs of OAC, enucleation and enucleation with systemic chemotherapy in 2012[28]. Based on their calculations, the costs associated with our average amount of OAC cycles (3.4 per patient in our center; unpublished results) would be $181,000 per patient. This is of course more than a simple enucleation treatment ($48.000) but less than the costs associated with systemic chemotherapy plus planned enucleation ($281.000).

For patients with unilateral disease who were almost always enucleated in the 20^th^ century our enucleation rate (for both naive and those who fail first line therapy) is 7.4%.

Despite the fact that OAC has been used for these advanced eyes, patient survival has not been compromised. To date no child we have treated with OAC has died of metastatic disease. This striking observation has been replicated in other centers worldwide[18,21,22,29–31].

OAC represents a profound change in retinoblastoma management resulting in shorter treatment times, lower morbidity, lower cost (in some centers), saving more eyes (many with vision) without compromising patient survival. It has reversed the 100-year-old treatment of retinoblastoma; in our center enucleation is no longer the most common treatment for unilateral retinoblastoma.

## Supporting Information

S1 Fig ICRb class of primary enucleated versus OAC treated eyes. The percentage of eyes that were ICRb group D (black) or ICRb group E (gray) are depicted per time period in OAC treated and primary enucleated eyes. The distribution of the number of D versus E eyes was compared to calculate statistical significance with Fisher’s exact test between different time periods and treatments. Significant differences in were marked with asterisks. * p = 0.0016, ** p<0.0001, *** p<0.0001. The number of eyes are listed underneath the figure (as opposed to the percentages in the bar graph).
(TIFF)

S2 Fig Primary enucleated versus OAC treated eyes per time period in D and E eyes. (A) The percentage of D eyes that were primary enucleated (black) or OAC treated (gray) in 2006–2009 versus 2010–2014. (B) As (A) but than in E eyes.
The distribution of the number of D and E eyes was compared between the two time periods (Fisher’s exact test). Significantly more E eyes were OAC treated versus enucleated in the period 2010–2014 (* p<0.0001).
(TIFF)
