# Supplementary figures and images for: Advanced Unilateral Retinoblastoma: The Impact of Ophthalmic Artery Chemosurgery on Enucleation Rate and Patient Survival at MSKCC
Source: PLoS One. 2015 Dec 28;10(12):e0145436. doi: 10.1371/journal.pone.0145436 (PMC4692433; doi:10.1371/journal.pone.0145436)

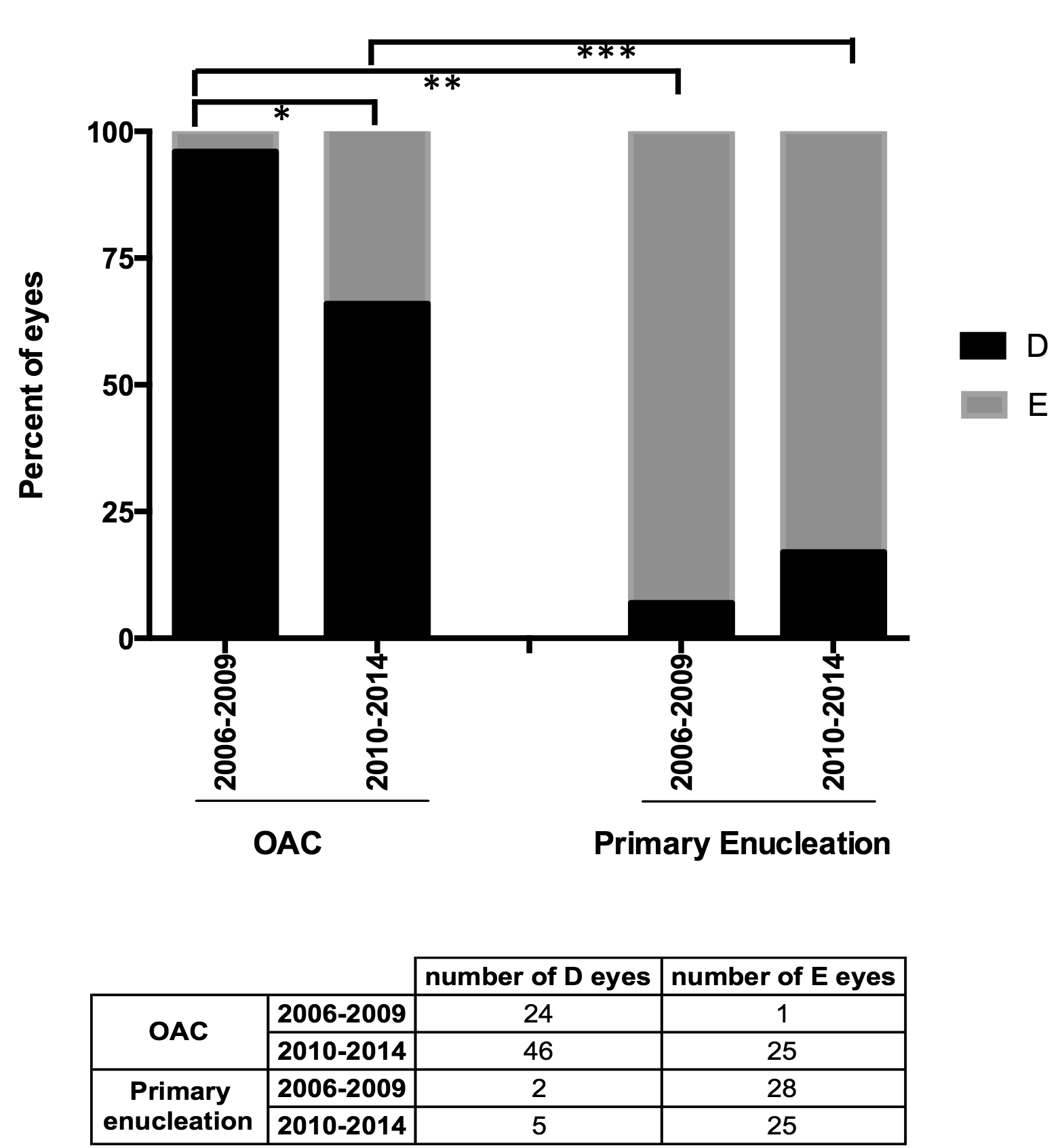

Supplement: S1 Fig — The percentage of eyes that were ICRb group D (black) or ICRb group E (gray) are depicted per time period in OAC treated and primary enucleated eyes. The distribution of the number of D versus E eyes was compared to calculate statistical significance with Fisher’s exact test between different time periods and treatments. Significant differences in were marked with asterisks. * p = 0.0016, ** p<0.0001, *** p<0.0001. The number of eyes are listed underneath the figure (as opposed to the percentages in the bar graph). (TIFF) [file pone.0145436.s001.tiff]

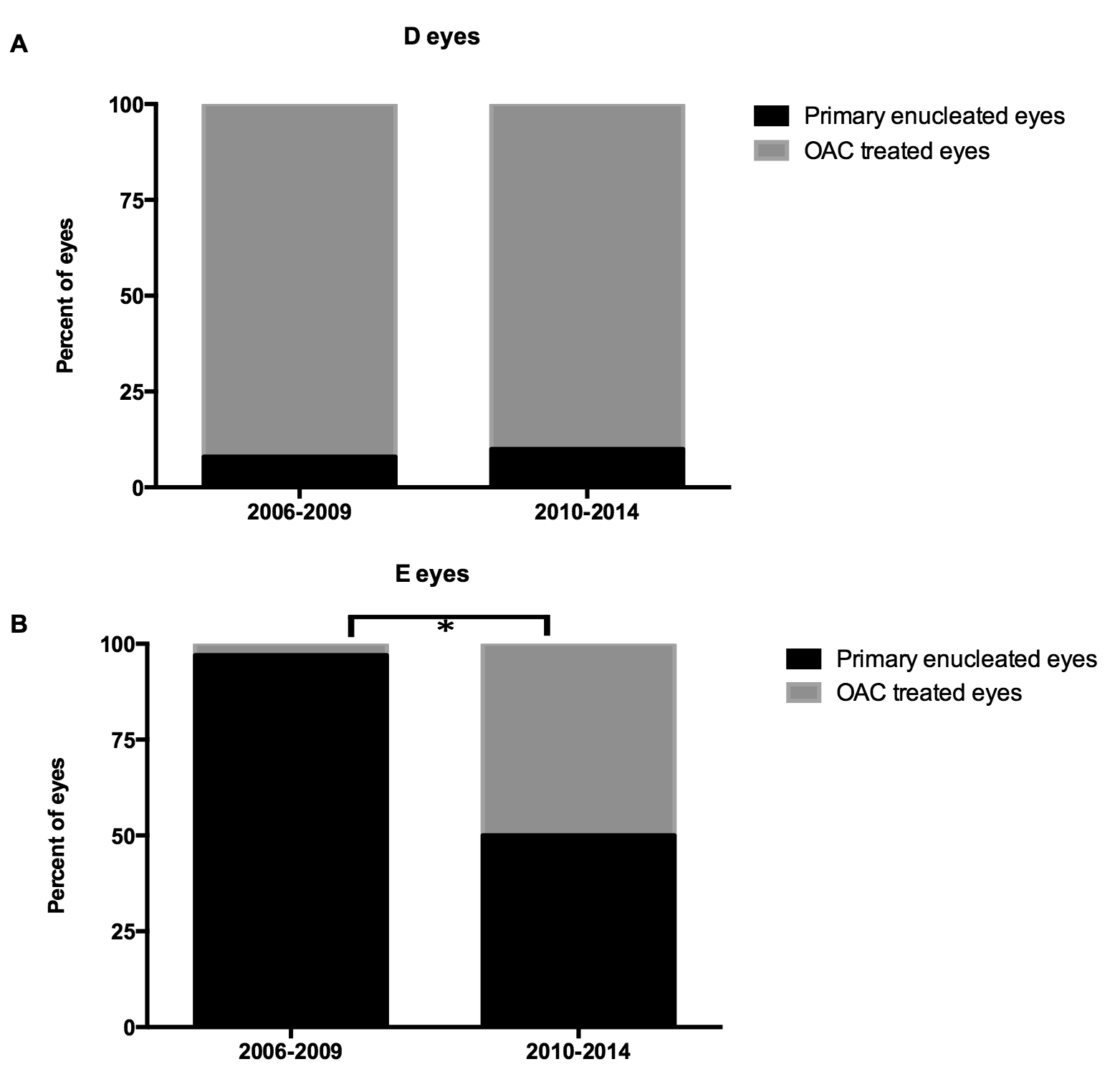

Supplement: S2 Fig — (A) The percentage of D eyes that were primary enucleated (black) or OAC treated (gray) in 2006–2009 versus 2010–2014. (B) As (A) but than in E eyes. The distribution of the number of D and E eyes was compared between the two time periods (Fisher’s exact test). Significantly more E eyes were OAC treated versus enucleated in the period 2010–2014 (* p<0.0001). (TIFF) [file pone.0145436.s002.tiff]
